# Microvesicles Derived from Human Bronchial Epithelial Cells Regulate Macrophage Activation During *Mycobacterium abscessus* Infection

**DOI:** 10.1021/acs.jproteome.4c00827

**Published:** 2025-03-28

**Authors:** Carlyn M. Guthrie, Amber C. Meeker, Ashton E. Self, Aidaly Ramos-Leyva, Olivia L. Clark, Stephen K. Kotey, Steven D. Hartson, Yurong Liang, Lin Liu, Xuejuan Tan, Yong Cheng

**Affiliations:** † Department of Biochemistry and Molecular Biology, Oklahoma State University, Stillwater, Oklahoma 74078, United States; ‡ Oklahoma Center for Respiratory and Infectious Diseases, Oklahoma State University, Stillwater, Oklahoma 74078, United States; § Department of Physiological Sciences, Oklahoma State University, Stillwater, Oklahoma 74078, United States; ∥ Center for Genomics and Proteomics, Oklahoma State University, Stillwater, Oklahoma 74078, United States

**Keywords:** extracellular vesicles, microvesicles, epithelial cells, macrophages, *Mycobacterium abscessus*, proteomics, intercellular communication

## Abstract

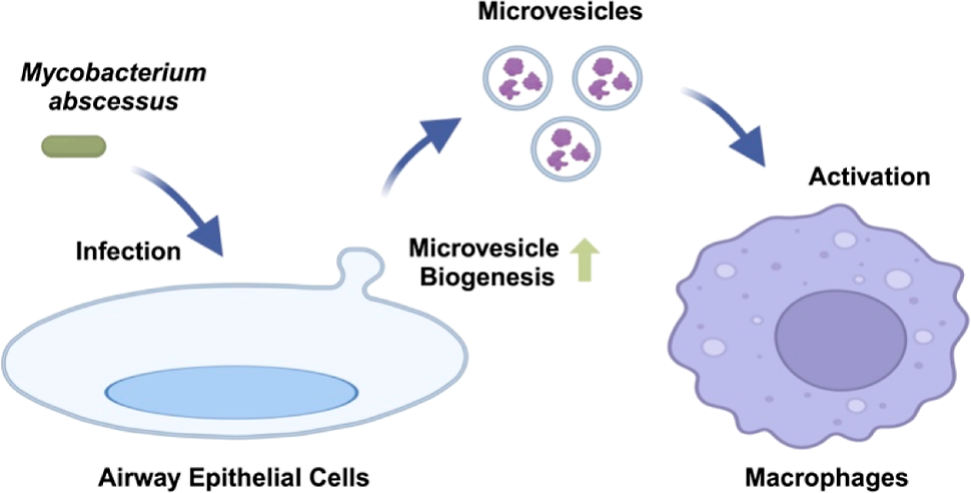

Intercellular communication is important for host immunity in response to bacterial infections. Nontuberculous mycobacterium (NTM), such as *Mycobacterium abscessus* (*M. ab*), is a group of environmental bacteria that can cause severe lung infections in individuals with pre-existing lung conditions, including cystic fibrosis (CF) and chronic obstructive pulmonary disease (COPD). There is limited knowledge understanding the interaction between airway epithelial cells and immune cells during NTM infections. In this study, we characterized microvesicles (MVs) released from uninfected and *M. ab*-infected human bronchial epithelial cells and investigated the effect of these MVs on the activation and polarization of THP-1-derived macrophages in cell culture. Our results indicate that MVs released by *M. ab*-infected human bronchial epithelial cells stimulated the activation of M2-polarized macrophages in cell culture when compared to MVs released by uninfected cells. Additionally, the proteomic analysis for isolated MVs showed that the proteins involved in the cell adhesion pathway were enriched in MVs from *M. ab*-infected human bronchial epithelial cells compared to MVs from uninfected cells. Among those, the cell surface protein, intercellular adhesion molecule 1 (ICAM-1), regulated the uptake of MVs released by *M. ab*-infected human bronchial epithelial cells by recipient macrophages in cell culture. In conclusion, our data suggest that in response to *M. ab* infection, human airway epithelial cells release MVs to modulate the activation of macrophages, which are key cells for mycobacterial intracellular survival in the host.

## Introduction

Nontuberculous mycobacteria (NTM) are a group of environmental bacteria and are generally nonpathogenic. However, they can cause severe lung infections in immunocompromised individuals or those with pre-existing lung conditions such as cystic fibrosis (CF) and chronic obstructive pulmonary disease (COPD).^1−3^*Mycobacterium abscessus* (*M. ab*) is one of the most common NTM species identified in CF patients with lung diseases. *M. ab* has evolved the ability to infect and survive within macrophages, which are immune cells responsible for engulfing and destroying invading pathogens in the immune system. Once inside macrophages, *M. ab* can manipulate the host cellular defense mechanisms, enabling them to avoid destruction and persist within host cells.^4,5^ This intracellular lifestyle allows *M. ab* to evade host immunity and contributes to mycobacterial resistance to antibiotic treatment. The intracellular survival of *M. ab* within macrophages also plays a role during the course of *M. ab* chronic infections, particularly in individuals with underlying health conditions such as CF.^6^ Therefore, it becomes important to understand how *M. ab* interacts with macrophages in the airway of patients or animal models, facilitating the development of more effective therapies for treating *M. ab* infections. It was recently found that *M. ab* can infect airway epithelial cells, the main cell type lining the surfaces of the respiratory tract, in cell culture.^7,8^ This suggests that the consequence of *M. ab* lung infection depends on multiple factors beyond airway macrophages, including an intercellular communication between airway epithelial cells and macrophages that has been understudied.

Microvesicles (MVs), one type of extracellular vesicles (EVs), are small membrane-bound vesicles released by a variety of cell types, including epithelial cells, immune cells, and cancer cells. These vesicles range in size from about 100 to 1000 nm in diameter and contain various biological molecules such as proteins, lipids, and nucleic acids. MVs play an important role in cell-to-cell communication by transferring their contents from donor cells to recipient cells, which subsequently can regulate cellular pathways and functions within these cells.^9,10^ MVs have been implicated in various physiological and pathological processes, including the host–pathogen interactions, immune responses, tissue repair, and the progression of diseases such as cancers. Additionally, MVs are being studied as potential biomarkers for human diseases and as therapeutic delivery systems due to their ability to carry bioactive cargo and their stability in the body.^9,10^ In the context of infectious diseases, MVs from host cells infected with microbial pathogens not only carry cargo from the host but also proteins, lipids and nucleic acids from the pathogens. These vesicles are critical components in host responses to invading pathogens and may play a detrimental or beneficial effect on host defense.^10^ Understanding the interactions between MVs and *M. ab* infection can provide insight into the mechanisms of disease progression and potentially lead to the development of new diagnostic and therapeutic strategies.

In this study, we investigated the effect of MVs released by uninfected and *M. ab*-infected human bronchial epithelial cells on macrophage activation and *M. ab* survival within THP-1-derived macrophages in cell culture. We also analyzed the protein profile of MVs isolated from uninfected and *M. ab*-infected human epithelial cells in cell culture. Our results indicate that MVs released by *M. ab*-infected epithelial cells increased the polarization of M2 macrophages in cell culture, and the MV-carried ICAM-1 (intercellular adhesion molecule 1) regulates the uptake of MVs released by *M. ab*-infected human bronchial epithelial cells by THP-1-derived macrophages in cell culture.

## Materials and Methods

### Mammalian Cell Culture

16HBE14o-human bronchial epithelial cell line was cultured at 37 °C and 5% CO_2_ in minimum essential medium (MEM) (Cat. no. SH30265; HyClone) supplemented with 10% (v/v) fetal bovine serum (Cat. no. 10438-026; Gibco) and streptomycin and penicillin (Cat. no. SV30010; HyClone) at a final concentration of 100 U/ml. THP-1 cell line was grown at 37 °C and 5% CO_2_ in RPMI 1640 medium (Cat. no. SH30027; HyClone) supplemented with 10% (v/v) fetal bovine serum. The study involving 16HBE14o- and THP-1 cell lines was approved by Oklahoma State University Institutional Review Board (IRB-23-433).

### Bacterial Culture

*M. ab* ATCC 19977 was grown to mid log phase in Middlebrook 7H9 broth (Cat. no. M198; HiMedia Laboratories) supplemented with 10% (v/v) OADC (oleic acid-albumin-dextrose-catalase, 0.05% Tween 80) at 37 °C and 5% CO_2_. Before use, mycobacterial culture was passed through a syringe fitted with a 27-gauge needle to interrupt bacterial clumps as we did previously.^11,12^

### MV Isolation and NanoSight Analysis

16HBE14o-human bronchial epithelial cells were uninfected or infected with *M. ab* at an MOI (multiplicity of infection) of 5 for 4 h and then washed with prewarmed PBS three times to remove the extracellular *M. ab*. These cells were subsequently incubated in EV-free MEM complete medium for 72 h before MV isolation. EV-free MEM was prepared as we did previously.^13^ MVs were isolated as described previously.^14^ Briefly, the culture supernatant was centrifuged at 1000 xg and 4 °C for 15 min to remove cells and debris. The supernatant was subsequently centrifuged at 2000 xg and 4 °C for 20 min to remove apoptotic bodies. Finally, human bronchial epithelial cell-derived MVs were spun down at 10,000 xg and 4 °C for 30 min. The MV pellets were washed with precold 1x PBS three times at 10,000 xg and 4 °C for 30 min and resuspended in 0.5 mL precold 1x PBS. Isolated MVs were analyzed by the NanoSight NS300 (Malvern Panalytical, UK) to determine the yield of MV isolation.

### Total RNA Isolation and Quantitative RT-PCR

Human THP-1-derived macrophages were first treated with phorbol 12-myristate 13-acetate (PMA, Cat. no. P1585; Sigma-Aldrich) for 24 h at 37 °C with 5% CO_2_, and then treated with MVs (Cell/MVs = 1:100) from uninfected or *M. ab*-infected human bronchial epithelial cells in vitro for 24 h. Total cellular RNA was isolated using Monarch’s Total RNA Miniprep Kit (Cat. no. T20105; New England Biolabs) according to manufacturer instruction. Quantitative PCR was performed as we did previously^15^ on the Roche LightCycler 480 real-time PCR system (Cat. no. 05815916001) with Luna Universal Master Mix (Cat. no. M3003E; New England Biolabs) and the primers for *TNF*-α, *IL*-1β, *IL-6*, *IL-10*, *ARG-1* and *GAPDH* as shown in Table 1.

**Table 1:** Primers in the Study

primer name	primer sequence 5′ → 3′
TNF-α forward	GCT GCA CTT TGG AGT GAT CG
TNF-α reverse	CAT GGG CTA CAG GCT TGT CA
IL-1β Forward	CAG GGA CAG GAT ATG GAG CA
IL-1β reverse	CAC GCA GGA CAG GTA CAG AT
IL-6 forward	AAC CAG TGG CTG CAG GAC AT
IL-6 reverse	CAT TAA CAA CAA CAA TCT GAG GT
IL-10 forward	AGT TTT ACC TGG AGG AGG TGA T
IL-10 reverse	GTT TTC ACA GGG AAG AAA TCG AT
ARG-1 forward	ACT CTA GGC ATT AAA TAC TTT TCA
ARG-1 reverse	ATG GGT CCA GTC CGT CAA CAT
*GAPDH* forward	GAA GGT GAA GGT CGG AGT CAA C
*GAPDH* reverse	CAT GGG TGG AAT CAT ATT GGA A

### Proteomic Analysis for MVs

Purified MV samples were processed as we did previously^6,15^ in the proteomics core facility at Oklahoma State University in Stillwater, OK. Raw data were analyzed against a database consisting of 80,581 human proteins (UniProt database UP000005640; *Homo sapiens*) and 4940 *M. ab* proteins (UniProt database UP000007137) using Perseus (version 2.0.10.0). The following default options in MaxQuant were used: (i) fixed modification: carbamidomethyl of cysteine (Cys), and (ii) variable modifications: oxidation of methionine (Met), acetylation of protein N-termini, and chemical cyclization of glutamine (Gln) residues to pyroglutamate if they are at the n-terminus of the peptide. For human proteins, the LFQ intensities of measured proteins were log_2_ transformed, and the proteins exhibiting a fold change >2 and *p* < 0.05 were considered to be differentially enriched. Protein pathway analysis was then conducted to elucidate the biological functions of the differentially regulated proteins using Metascape custom analysis- *H. sapiens* GO: biological processes, as we did previously.^6,15,16^ For *M. ab* proteins, the pathway analysis was performed using KEGG mapper as we described previously.^12^

### *Mycobacterium abscessus* Survival Assay in THP-1-derived Macrophages

THP-1 cells were preseeded at a density of 2.5 × 10^4^ cells/well in 96-well TC-treated plates (Corning Costar, Cat. no. 07-200-90) in the presence of 25 ng/mL PMA for 24 h at 37 °C with 5% CO_2_. PMA-treated cells were then treated with MVs isolated from uninfected or *M. ab*-infected 16HBE14o-human bronchial epithelial cells for 24 h. Treated cells were infected with *M. ab* (MOI = 5) for 1 h and then washed three times with RPMI media to remove extracellular bacteria. Cells were incubated for an additional 1, 24, and 72 h before lysing and plating on Middlebrook 7H10 agar (Himedia, Cat. no. M199) plates and incubating at 37 °C as we did previously.^17^

### MV Uptake Assay in THP-1-Derived Macrophages

THP-1 cells were first treated with PMA for 24 h at 37 °C with 5% CO_2_ as described above. Treated cells were then untreated or treated with dye-labeled MVs (Cell/MVs = 1:100) in vitro for 24 h at 37 °C with 5% CO_2_, and visualized using Invitrogen EVOS M5000 Imaging System. MVs were labeled with Alexa Fluor 555-conjugated WGA (Cat. NoW32464; invitrogen) as described previously.^17^ Before the MV treatment, MVs were pretreated with mouse antihuman ICAM-1 antibody (Cat. no. 322702; BioLegend) or mouse IgG isotype control (Cat. no. 400166; BioLegend) for 30 min at room temperature.

## Results

### Characterization of MVs Released by Uninfected or *M. ab*-Infected Human Bronchial Epithelial Cells

**Figure 1:**
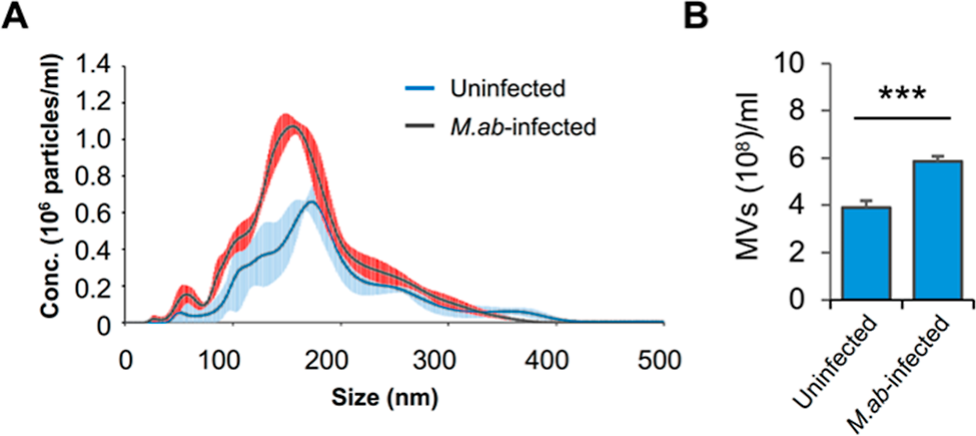
Characterization of purified MVs. (A) NanoSight analysis for MVs isolated from uninfected (uninfected MVs) or *M. ab-*infected (*M. ab*-infected MVs) human bronchial epithelial cells. Solid line indicates average number of particles (*n* = 5). Red and light blue shading demonstrates standard deviation for each sample. (B) Microvesicle yield from uninfected or *M. ab-*infected human bronchial epithelial cell culture. Total microvesicle yield was determined by NanoSight NS300 and values were calculated based on the volume of cell culture used for vesicle isolation. Data are representative of three independent experiments. The results in (B) are mean ± SD (*n* = 3/group). ***p < 0.001 by two-tailed Student’s *t*-test.

To investigate the role of MVs in the cell-to-cell communication between airway epithelial cells and macrophages, we isolated MVs from uninfected or *M. ab*-infected 16HBE14o-human bronchial epithelial cells in cell culture. As seen in Figure 1A, NanoSight analysis revealed that *M. ab* infection slightly affects the size distribution pattern of released MVs when compared to uninfected human bronchial epithelial cells. A smaller peak was observed in MVs from *M. ab*-infected human bronchial epithelial cells relative to MVs from uninfected cells. Additionally, our results indicate that *M. ab*-infected human bronchial epithelial cells released more MVs than uninfected human bronchial epithelial cells under experimental conditions in cell culture (Figure 1B).

### MVs Released by *M. ab*-Infected Human Bronchial Epithelial Cells Regulate the Expression of M1/M2 Markers in THP-1-derived Macrophages in Cell Culture

**Figure 2:**
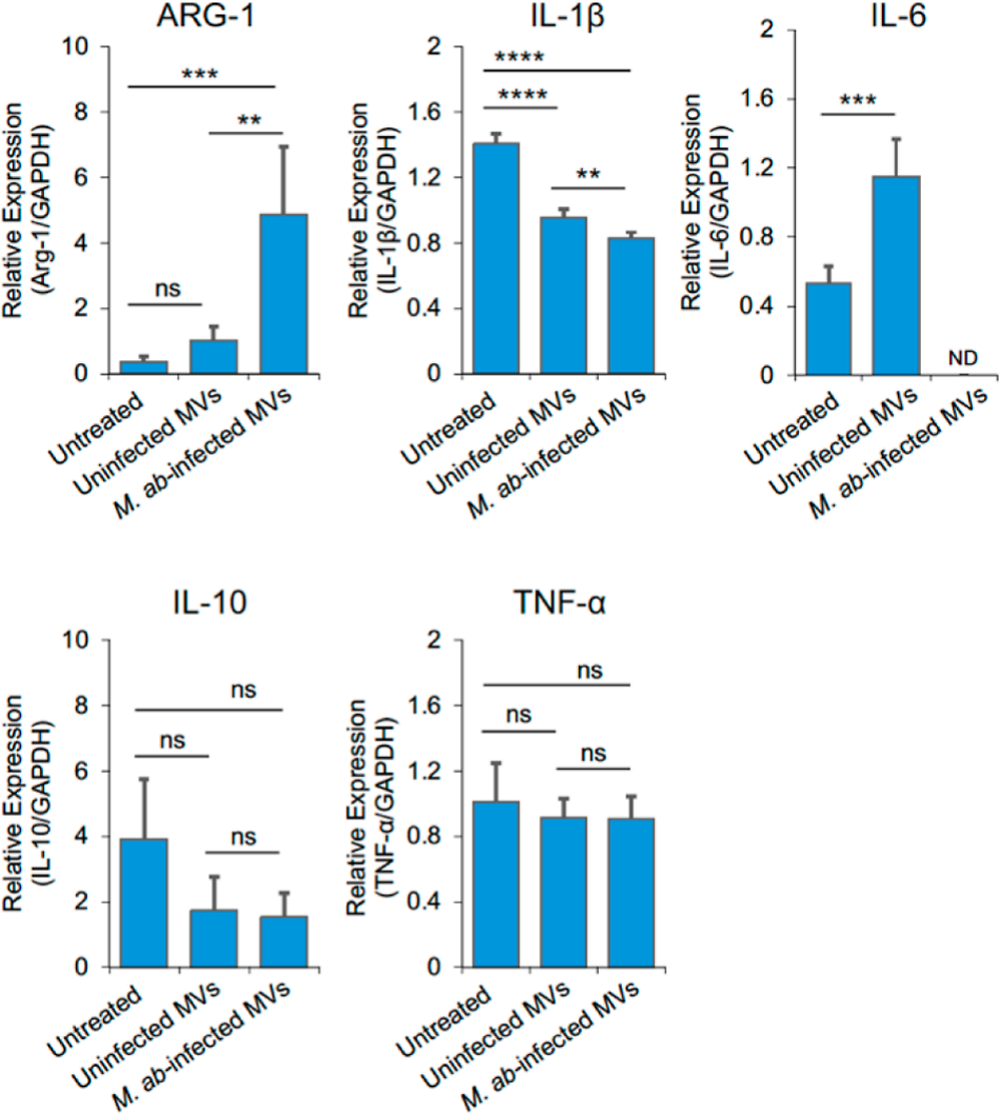
Quantitative RT-PCR for M1-or M2-macrophage marker genes. Human THP-1-derived macrophages were untreated or treated with MVs isolated from uninfected (uninfected MVs) or *M. ab-*infected (*M. ab*-infected MVs) human bronchial epithelial cells for 24 h at a ratio of 100 (MVs: cell). The mRNA levels of *ARG-1*, *IL*-1β, *IL-6*, *IL-10* and *TNF*-α were normalized to *GAPDH* and expressed a fold change relative to the uninfected MVs group. The results are representative of three independent experiments. The data are mean ± SD (*n* = 3/group). ND, not detected. n.s., not significant; **p < 0.01, ***p < 0.001 and ****p < 0.0001 by one-way ANOVA, followed by Tukey’s post hoc test.

**Figure 3:**
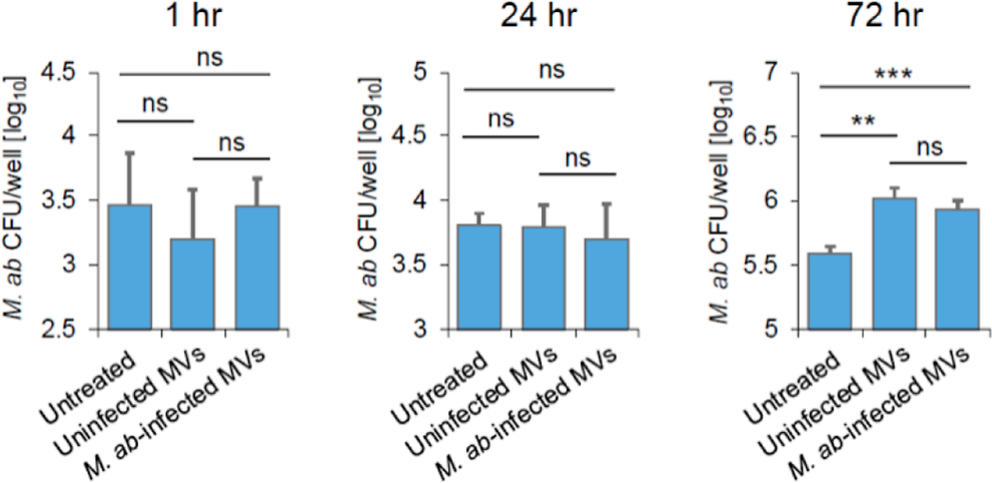
*M. ab* survival assay in macrophages. THP-1-derived macrophages were infected with *M. ab* in the presence of MVs isolated from uninfected (uninfected MVs) or *M. ab*-infected (*M. ab*-infected MVs) human bronchial epithelial cells (MVs: cell = 100). The results are representative of three independent experiments. The data are mean ± SD (*n* = 3/group). n.s., not significant; **p < 0.01 and ***p < 0.001 by one-way ANOVA, followed by Tukey’s post hoc test.

To determine if human bronchial epithelial cell-derived MVs regulate human macrophage function, we measured the expression of M1 (*TNF*-α, *IL-6* and *IL*-1β) and M2 (*ARG-1* and *IL-10*) macrophage markers by quantitative RT-PCR in THP-1-derived macrophages that were treated for 24 h with MVs released by uninfected or *M. ab*-infected human epithelial cells in cell culture. As shown in Figure 2, MVs released by *M. ab*-infected human bronchial epithelial cells significantly upregulated the expression of *ARG-1* in THP-1-derived macrophages in cell culture when compared to MVs released by uninfected human bronchial epithelial cells. In contrast, the expression of M1 markers *IL*-1β and *IL-6* were significantly downregulated by MVs released by *M. ab*-infected human bronchial epithelial cells compared to MVs from uninfected human bronchial epithelial cells. No difference was detected for the expression of *TNF*-α and *IL-10* in THP-1-derived macrophages that were untreated or treated with MVs from uninfected or *M. ab*-infected human bronchial epithelial cells. We also measured *M. ab* intracellular survival within THP-1-derived macrophages with or without MVs in cell culture. Our results indicate that MVs from either uninfected or *M. ab*-infected human bronchial epithelial cells had no significant effect on *M. ab* intracellular survival within THP-1-derived macrophages at 1 and 24 h post *M. ab* infection (Figure 3). However, when compared to untreated cells, MVs from either uninfected or *M. ab*-infected human bronchial epithelial cells significantly increased *M. ab* intracellular survival in THP-1-derived macrophages at 72 h post *M. ab* infection. No difference was detected for *M. ab* survival in THP-1-derived macrophages that were treated with MVs from uninfected or *M. ab*-infected human bronchial epithelial cells in each time point (Figure 3).

### Proteomic Analysis for MVs Released by Uninfected or *M. ab*-Infected Human Bronchial Epithelial Cells in Cell Culture

**Figure 4:**
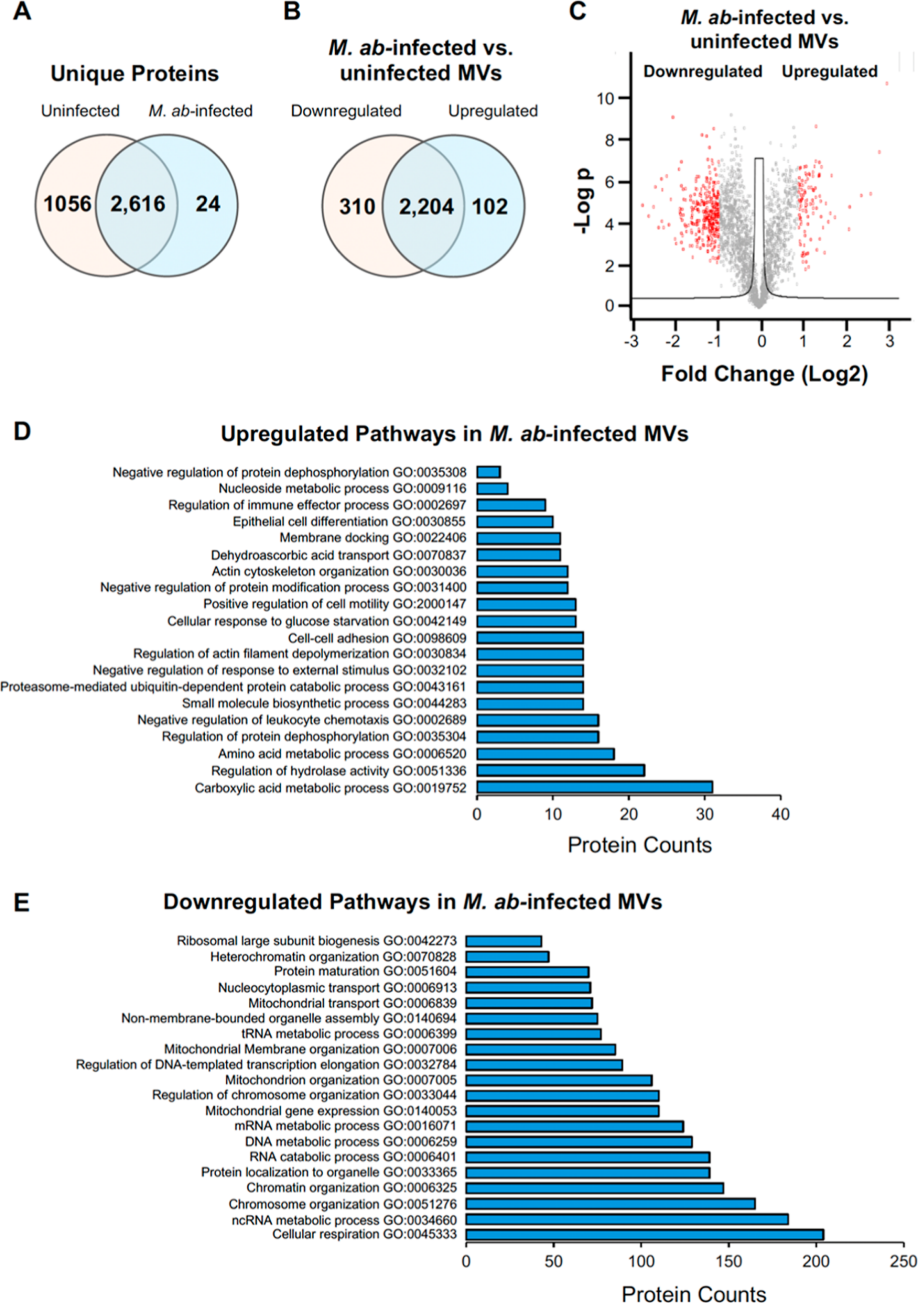
Proteomic analysis for purified MVs. (A) Venn diagram for human proteins identified in MVs isolated from either uninfected or *M. ab*-infected human bronchial epithelial cells. (B) Venn diagram for overlapping human proteins that are differentially enriched in MVs isolated from uninfected and *M. ab*-infected human bronchial epithelial cells (cutoff: fold change >2 and *p* < 0.05). (C) Volcano plot for human proteins differentially enriched in MVs isolated from uninfected or *M. ab*-infected human bronchial epithelial cells. (D) Metascape pathway analysis for proteins that were upregulated in MVs isolated from *M. ab*-infected human bronchial epithelial cells compared to those from uninfected epithelial cells. (E) Metascape pathway analysis for proteins that were downregulated in MVs isolated from *M. ab*-infected human bronchial epithelial cells.

To further characterize MVs released by epithelial cells, we determined the proteomic profile of MVs released by uninfected or *M. ab*-infected human bronchial epithelial cells as we did previously.^15^ As shown in Figure 4A and Supporting Information Table S1, we identified 24 unique human proteins in MVs from *M. ab*-infected human bronchial epithelial cells, and 1056 unique human proteins in MVs from uninfected human bronchial epithelial cells. Among 2616 overlapping human proteins that were detected in MVs released by either uninfected or *M. ab*-infected human bronchial epithelial cells (Figure 4A), the abundance of 102 proteins (Figure 4B and Supporting Information Table S2) was upregulated in MVs from *M. ab*-infected human bronchial epithelial cells. The abundance of 310 proteins were downregulated (Figure 4B, Supporting Information Table S2) when compared to MVs from uninfected human bronchial epithelial cells. 2204 proteins had a comparable abundance in MVs from both conditions (Supporting Information Table S2). In Figure 4C, a volcano plot highlighted the differentially enriched proteins as shown in Figure 4B.

**Figure 5:**
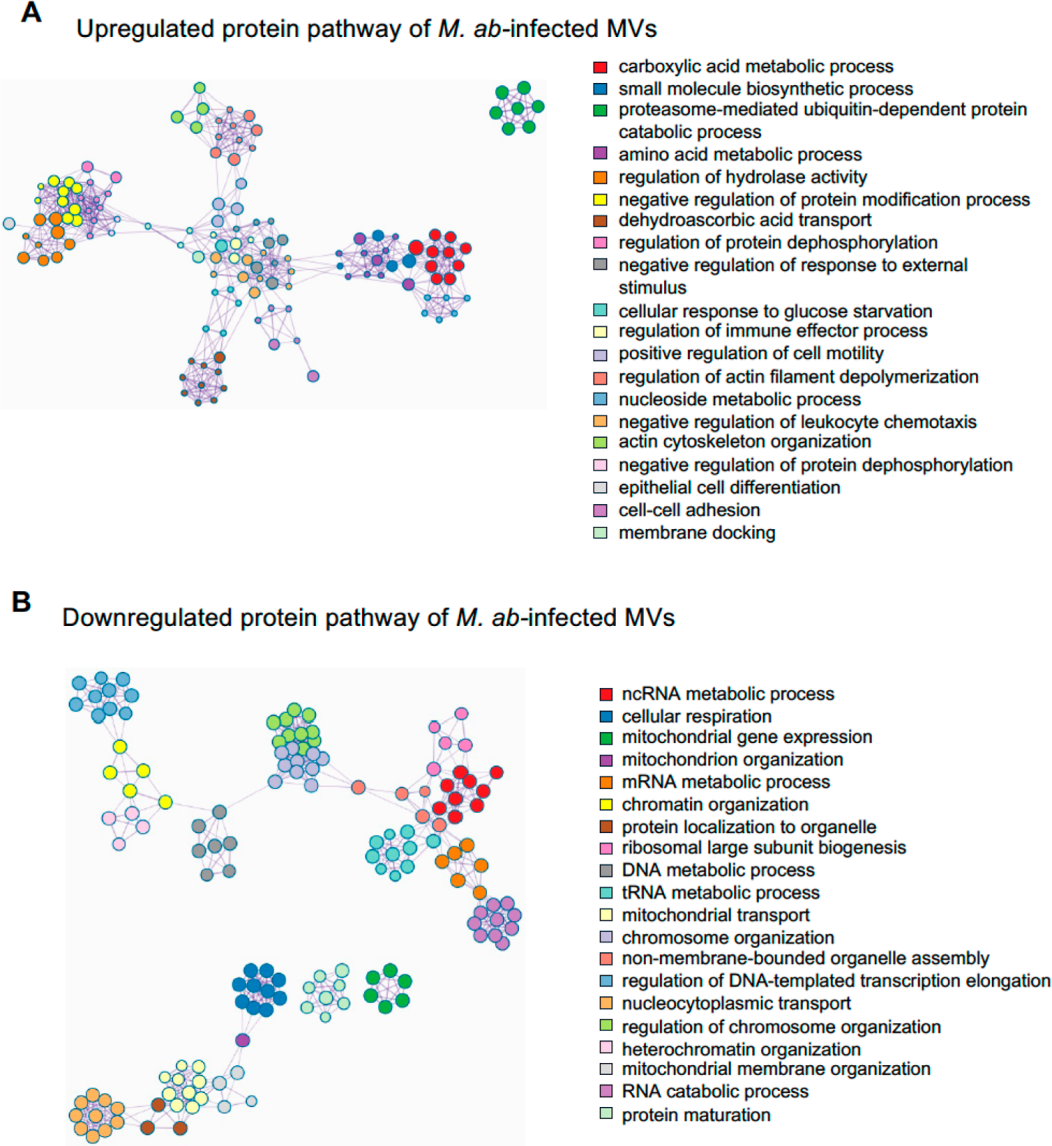
Metascape network for enriched ontology clusters. The analysis was performed using human proteins that were differentially enriched in MVs isolated from *M. ab*-infected human bronchial epithelial cells compared to those from uninfected human bronchial epithelial cells. (A) Metascape network for enriched ontology clusters based on the upregulated proteins in MVs isolated from *M. ab*-infected human bronchial epithelial cells (related to Figure 4D). (B) Similar to (A), but using the downregulated proteins in MVs isolated from *M. ab*-infected human bronchial epithelial cells (related to Figure 4E). Each term is indicated by a circular node. The number of input proteins falling into that term is represented by the circle size, and the cluster identities are represented by colors.

To better understand the engagement of differentially enriched human proteins in MVs, we performed Metascape pathway analysis as we did previously.^15^Figure 4D shows the top 20 upregulated cellular pathways based on the enriched proteins in MVs from *M. ab*-infected human bronchial epithelial cells, including carboxylic acid metabolic progress (GO:0019752), regulation of hydrolase activity (GO:0051336), amino acid metabolic process (GO:0006520), regulation of protein dephosphorylation (GO:0035304), negative regulation of leukocyte chemotaxis (GO:0002689), small molecule biosynthetic process (GO:0044283), proteasome-mediated ubiquitin-dependent protein catabolic process (GO:0043161), negative regulation of response to external stimulus (GO:0032102), regulation of actin filament depolymerization (GO:0030834), and cell–cell adhesion (GO:0098609). The gene lists of the top 20 enriched pathways are shown in Supporting Information Table S3. Conversely, we identified more downregulated cellular pathways based on enriched proteins in MVs released by *M. ab*-infected epithelial cells. Figure 4E shows the top 20 downregulated cellular pathways according to Metascape pathway analysis, such as cellular respiration (GO:0045333), ncRNA metabolic process (GO:0034660), chromatin organization (GO:0006325), protein localization to organelle (GO:0033365), RNA catabolic process (GO:0006401), DNA metabolic process (GO:0006259), mRNA metabolic process (GO:0016071), mitochondrial gene expression (GO:0140053), and regulation of chromosome organization (GO:0033044). The gene lists of the top 20 downregulated cellular pathways are available in Supporting Information Table S4. Metascape network analysis for enriched ontology clusters shows that the top 20 upregulated pathways present a strong intercluster and intracluster similarity (Figure 5A), indicating a high degree of functional coherence among these pathways. This suggests that these pathways are closely related in terms of biological processes and molecular functions. A similar result was observed for the top 20 downregulated pathways in MVs released by *M. ab*-infected human bronchial epithelial cells relative to uninfected cells (Figure 5B).

### MVs Released by *M. ab*-Infected Human Bronchial Epithelial Cells in Cell Culture Carry Mycobacterial Proteins

**Figure 6:**
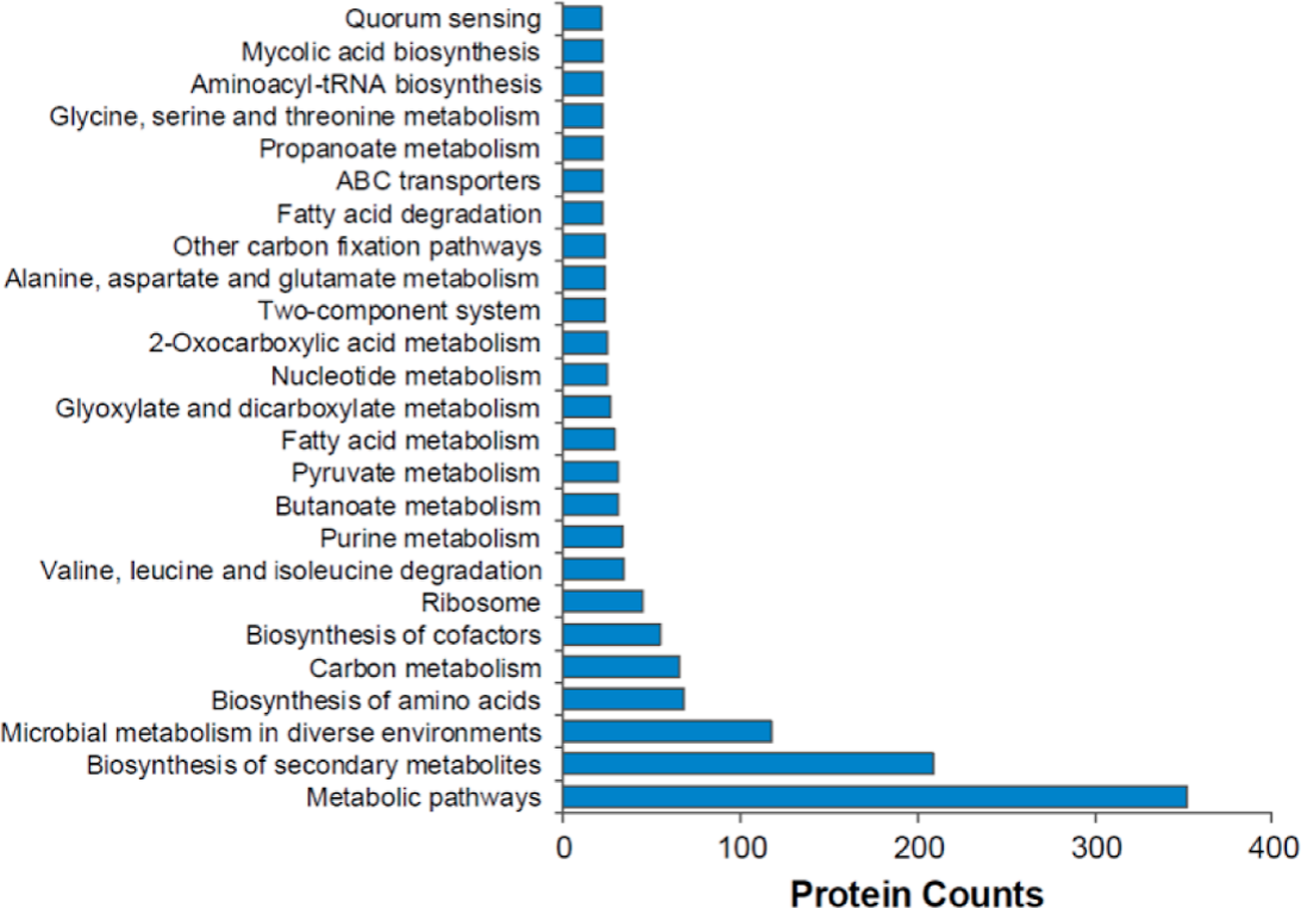
Metascape pathway analysis for *M. ab* proteins that were identified in MVs isolated from *M. ab*-infected human bronchial epithelial cells.

We also analyzed the profile of mycobacterial proteins in MVs isolated from *M. ab*-infected human bronchial epithelial cells in cell culture. As shown in Supporting Information Table S5, we identified 1264 mycobacterial proteins in MVs released by *M. ab*-infected human bronchial epithelial cells but absent in MVs isolated from uninfected human bronchial epithelial cells. Similar to EVs isolated from *Mycobacterium tuberculosis* (*M. tb*)-infected macrophages in cell culture or TB patients,^10^ a list of common mycobacterial proteins were found in MVs isolated from *M. ab*-infected human bronchial epithelial cells in cell culture in vitro, including Antigen 85 A/B/C (Ag85A/B/C), Chaperonin GroEL, Lipoproteins (such as lpqH, LpqW and LprC), Catalase-peroxidase CatG, Low molecular weight t-cell antigen tb8.4, Proteasome core protein PrcB, Alanine and proline-rich secreted protein Apa and Alanine dehydrogenase Ald (Supporting Information Table S5). The ESAT-6/EsxA and CFP10/EsxB proteins are two important components in the ESX-1 protein secretion system that plays a critical role in *M. tb* pathogenesis in the host but does not exist in *M. ab*.^19^ It was recently found that *M. ab* proteins EsxT (*MAB_3753c*) and EsxU (*MAB_3754c*) likely play a similar role as *M. tb* ESAT-6 and CFP10 protein in regulating phagolysosome maturation within host cells. Different from the EsxA and EsxB proteins in *M. tb*, EsxT and EsxU are secreted via an ESX-4 protein secretion system in *M. ab*.^20^ Interestingly, both EsxT and EsxU were identified in MVs from *M. ab*-infected human bronchial epithelial cells in cell culture (Supporting Information Table S5). We further performed pathway analysis for MV-carried *M. ab* protein as we did previously.^12^ As shown in Figure 6. 25 mycobacterial cellular pathways were enriched and each pathway covers at least 20 identified *M. ab* proteins. Among those, the *M. ab* proteins involved in the metabolic pathway were highly enriched in MVs from *M. ab*-infected human bronchial epithelial cells. The protein list for each enriched cellular pathway is shown in Supporting Information Table S6.

### MV-Carried ICAM-1 Facilitates the Uptake of MVs Released by *M. ab*-Infected Human Bronchial Epithelial Cells by THP-1-derived Macrophages in Cell Culture

**Figure 7:**
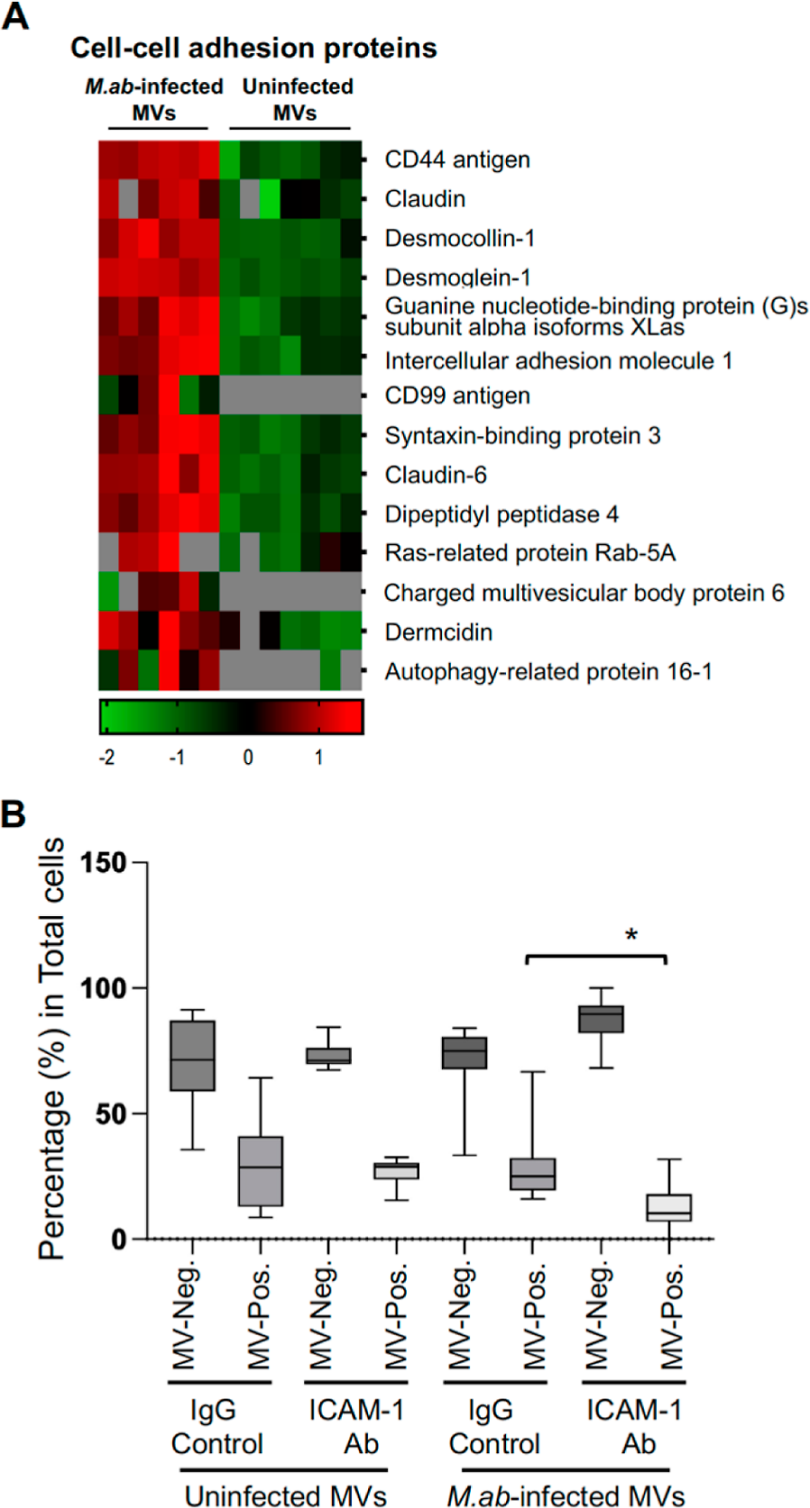
MV uptake assay in THP-1-derived macrophages. (A) Heatmap for the proteins involved in the cell–cell adhesion. (B) MV uptake assay in macrophages. THP-1-derived macrophages were treated with MVs isolated from uninfected (uninfected MVs) or *M. ab-*infected (*M. ab*-infected MVs) human bronchial epithelial cells for 24 h at a ratio of 100 (MVs: cells). Before the MV treatment, MVs were labeled with Alexa Fluor 555-conjuated WGA, and then pretreated with anti-ICAM-1 Ab (ICAM-1 Ab) or IgG isotype control (IgG control). MV-negative (MV-Neg.) and MV-positive (MV-Pos.) cells were visualized using fluorescence microscope. The results were expressed as a percentage of MV-negative or MV-positive cells relative to the total cells in each field. Data are mean ± SD (12 random fields/condition). *p < 0.05 by one-way ANOVA, followed by Tukey’s post hoc test.

As we described above, the cell–cell adhesion pathway was upregulated in MVs released by *M. ab*-infected human bronchial epithelial cells compared to the vesicles from uninfected cells (Figure 4D). A heatmap for enriched proteins involved in the cell–cell adhesion pathway is shown in Figure 7A. Interestingly, the protein abundance of ICAM-1 was much higher in MVs released by *M. ab*-infected human bronchial epithelial cells compared to the vesicles from uninfected cells (Figure 7A). ICAM-1 is a transmembrane glycoprotein that plays a crucial role in the immune response and is expressed on the surface of various cell types, including epithelial cells, endothelial cells and leukocytes. ICAM-1 serves as a ligand for the integrin leukocyte function-associated antigen 1 (LFA-1), which is expressed on the surface of immune cells such as macrophages.^17^ Therefore, we hypothesized that human macrophages might more efficiently uptake MVs released by *M. ab*-infected human bronchial epithelial cells, due to an increased level of MV-carried ICAM-1, compared to MVs released by uninfected epithelial cells in cell culture. Unexpectedly, a similar frequency of MV uptake was detected in THP-1-derived macrophages for MVs from either uninfected or *M. ab*-infected human bronchial epithelial cells (IgG control in Figure 7B). Interestingly, ICAM-1 blocking antibody significantly diminished the uptake of *M. ab*-infected MVs (released by *M. ab*-infected human bronchial epithelial cells) by THP-1-derived macrophages in cell culture. However, ICAM-1 blocking antibody had no effect on the uptake of uninfected MVs (released by uninfected human bronchial epithelial cells) by THP-1-derived macrophages in cell culture (Figure 7B).

## Discussion

Airway epithelial cells form the lining of the respiratory tract and play a vital role in respiratory function, defending against infections by serving as the first line of defense in the respiratory tract. These cells provide a barrier that prevents pathogens and harmful particles from entering deeper into lung tissues. Airway epithelial cells include several specialized cell types such as ciliated cells, goblet cells, and alveolar cells (type I and type II).^21^ During infection, airway epithelial cells can detect pathogen-associated molecular patterns (PAMPs) via pattern recognition receptors (PRRs) and release antimicrobial peptides, cytokines and chemokines, which recruit immune cells, such as macrophages, dendritic cells and neutrophils, to the site of infection and coordinate the immune response.^21^ However, some bacterial and viral pathogens, such as SARS-CoV-2 virus, have evolved the ability to hijack airway epithelial cells by attaching to and entering them, which can compromise the antimicrobial activity of epithelial cells.^22,23^ In some cases, this interaction between host cells and pathogens can trigger excessive inflammation and contribute to the severity of respiratory diseases such as pneumonia, influenza, and COVID-19.^21^ Understanding how airway epithelial cells interact with pathogens and immune cells is crucial for developing effective treatments and preventive strategies against infectious diseases of the respiratory system. There is very limited study investigating the interplay between airway epithelial cells and immune cells in response to NTM lung infection. NTM primarily infects alveolar macrophages which serve as host cells for mycobacterial replication in the host.^1,2^ Recently, it was found that *M. ab* could infect human bronchial epithelial cells in cell culture.^7,8^ This suggests a potential communication between *M. ab*-infected epithelial cells and macrophages, which may play a critical role in determining the consequence of *M. ab* lung infection.

Epithelial cells play a complex role in regulating macrophages that can either be detrimental to or beneficial to the host in response to microbial infections. As described above, during microbial infections, epithelial cells secrete various signaling molecules such as cytokines and chemokines, which attract and activate macrophages to the infection site. This helps coordinate the immune response and promote pathogen clearance.^21^ However, in chronic inflammatory diseases, such as CF or COPD, the interaction between epithelial cells and macrophages can exacerbate tissue damage and the progression of disease.^24^ Epithelial cells may produce pro-inflammatory mediators that sustain the activation of macrophages, leading to persistent inflammation and tissue destruction. Additionally, epithelial cells can influence the polarization of macrophages into either pro-inflammatory (M1) or anti-inflammatory (M2) phenotypes, which affects the balance between inflammation and tissue repair. An imbalance between these regulatory mechanisms can contribute to disease progression and chronic inflammation.^24^ Understanding these regulatory mechanisms offers potential therapeutic targets for modulating the immune response and improving outcomes in diseases involving chronic inflammation.

Besides cytokines and antimicrobial peptides as described above, airway epithelial cells also release extracellular vesicles, including exosomes and MVs, that carry active molecules from parental cells, including proteins, lipids and nucleic acids, that regulate macrophage migration and recruitment in the lung.^25,26^ It has been found that hyperoxia-induced oxidative stress increased MV release in human bronchial epithelial cell line BEAS-2B. These vesicles stimulate THP-1-derived macrophage migration and the expression of M1 macrophage marker genes *TNF*-α and *IL*-1β in THP-1-derived macrophages compared to MVs isolated from control cells.^25^ Similarly, MVs released from mouse lung epithelial cells were also induced by acid stress. Resulting MVs increased macrophage migration and activation as well.^26^ Altogether, it suggests airway stress is one of the main drivers to elevate extracellular vesicle release from epithelial cells and facilitates the crosstalk between epithelial cells and macrophages via extracellular vesicles. In contrast, little is known about the engagement of airway epithelial cell-derived extracellular vesicles in host responses to bacterial infections. In this study, we identified that the bacterial pathogen, *M. ab*, increased MV release from 16HBE14o-human bronchial epithelial cells in cell culture. Different from MVs from human BEAS-2B cells, another type of human bronchial epithelial cells, that were treated under hyperoxia, MVs from *M. ab*-infected 16HBE14o-human bronchial epithelial cells facilitate the expression of M2 macrophage marker gene *ARG-1* but attenuate M1 marker *IL*-1β and *IL-6* in human THP-1-derived macrophages in cell culture (Figure 2). It has been found previously that, relative to M1 macrophages, M2 macrophages are more permissive to mycobacterial intracellular survival and replication in cell culture.^27^ Unexpectedly, our results show that MVs from *M. ab*-infected human bronchial epithelial cells have no significant effect on *M. ab* intracellular survival within human macrophage in cell culture when compared MVs from uninfected human bronchial epithelial cells (Figure 3). Additional studies would be needed to address the effect of epithelial cell-derived MVs on macrophage activation and *M. ab* survival in the lung of mice. However, the interaction between epithelial cells and immune cells is more complex in vivo in mice or humans. The consequence of *M. ab* lung infection would be determined by multiple factors in which epithelial cell-derived MVs only play one role. It would be worthy to understand if macrophage-derived extracellular vesicles also regulate the activity of airway epithelial cells in the context of *M. ab* infection. Furthermore, future studies would also be needed to investigate if airway epithelial cell-derived MVs play similar roles in macrophages in response to other mycobacterial pathogens, such as *Mycobacterium avium* and *M. tb*.^17,28^

The uptake of extracellular vesicles by recipient cells is a complex process that involves specific interactions between the extracellular vesicle and the cell surface molecules, followed by internalization via endocytosis. For example, heparan sulfate proteoglycans (HSPGs) on recipient cell surfaces function as receptors of cancer cell-derived exosomes and regulate exosome uptake.^9,29^ Relative to exosomes, there is limited knowledge on MV uptake by recipient cells, especially in the context of infectious diseases. In this study, we found the ICAM-1 protein is enriched in MVs isolated from *M. ab*-infected human bronchial epithelial cells compared to MVs from uninfected cells (Figure 5A). Pretreatment of MVs using an ICAM-1 blocking antibody significantly diminished the *M. ab*-infected MV internalization by human THP-1-derived macrophages in cell culture (Figure 5B). Therefore, our results suggests that MV-carried ICAM-1 potentially serves as a receptor for epithelial cell-derived MV uptake by macrophages in the context of *M. ab* infection. It has been well-known that ICAM-1 is a cell surface glycoprotein that plays a critical role in cell–cell adhesion by binding to Mac-1 (macrophage adhesion ligand-1) and LFA-1 that are expressed in either human and mouse macrophages.^17^ Therefore, our data further suggests that ICAM-1 potentially regulates human bronchial epithelial cell-derived MV internalization by macrophages via recognizing cell surface protein Mac-1 and/or LFA-1, during *M. ab* infection. In future studies, it would be worthy to further understand the interactions between MV-carried ICAM-1 and macrophage surface proteins, Mac-1 and/or LFA-1, and their effect on MV uptake by macrophages. These experiments could be performed using bone marrow-derived macrophages isolated from *CD11b*^–/–^ or *CD11a*^–/–^ mice. CD11b and CD11a are subunits of Mac-1 and LFA-1, respectively, in mice.^30^ Our results also showed that blocking ICAM-1 did not interfere with uninfected epithelial cell-derived MV internalization by macrophages (Figure 7B). This suggests that other pathways or MV-carried molecules, such as heparan sulfate proteoglycans as described above,^9,29^ are likely involved in uninfected human bronchial epithelial cell-derived MV internalization by THP-1-derived macrophages in cell culture.

Other cell–cell adhesion molecules appear to be upregulated in MVs isolated from *M. ab*-infected 16HBE14o-human bronchial epithelial cells in cell culture (Figure 7A). CD44 and CD99 are two well-known cell surface proteins that have been identified in EVs from various types of mammalian cells.^31,32^ It was recently reported that pancreatic cancer cell-released exosomes regulate the function of liver cells via exosome-carried CD44 that interacts with integrin α6β4 on recipient cell surface.^31^ Tight junctions are a key structural component of epithelial cells and assist with cell–cell adhesion mechanisms.^33,34^ Claudin, claudin-6, desmocollin-1, desmoglein-1 proteins were shown to be enriched in MVs isolated from *M. ab*-infected 16HBE14o-human bronchial epithelial cells in cell culture when compared to MVs released by uninfected cells (Figure 7A). These proteins are well associated with epithelial cell tight junction formation and cell-to-cell interactions.^33,34^ This raises the question of whether MVs released by *M. ab*-infected 16HBE14o-human bronchial epithelial cells could potentially regulate the formation of tight junctions in airway epithelium. In future studies, it would be important to investigate if airway epithelial cells mediate tight junction dynamics via releasing MVs in response to *M. ab* infection in animal models and humans. Besides the proteins described above, we also found that three proteins, syntaxin-binding protein 3 (STXBP3), ras-related protein Rab-5A (RAB5A) and charged multivesicular body protein 6 (CHMP6), were highly enriched in MVs isolated from *M. ab*-infected 16HBE14o-human bronchial epithelial cells compared to MVs released by uninfected cells in cell culture (Figure 7A). Interestingly, these proteins or their family members have been demonstrated to regulate the biogenesis of extracellular vesicles, exosomes or MVs, in mammalian cells in cell culture.^6,10,35^ It would be worthy to determine if these proteins also regulate and/or increase MV biogenesis in 16HBE14o-human bronchial epithelial cells in response to mycobacterial infection in the future study (Figure 1B).

Previous studies indicate that macrophage-released EVs, such as exosomes, carry mycobacterial proteins in response to mycobacterial infection, including *M. tb* and *M. avium*.^10,13,36^ These exosome-carried mycobacterial proteins are excellent candidates as novel biomarkers for mycobacterial infection. Additionally, some exosome-carried mycobacterial proteins act as PAMPs that can be detected by the host PRRs in immune cells, such as macrophages, dendritic cells and neutrophils, ultimately inducing innate and adaptive immune response.^37^ Similarly, the proteomic analysis in our study identified a list of *M. ab* proteins in MVs released by *M. ab*-infected human bronchial epithelial cells in cell culture (Supporting Information Table S5). Interestingly, we found that mycobacterial proteins involved in specific cellular pathways, such as the metabolic pathway, are highly enriched or trafficking to MVs released by *M. ab*-infected human bronchial epithelial cells (Figure 6 and Supporting Information Table S6). Different from exosomes or MVs released by macrophages in response to mycobacterial infection,^10^ we know little about exosome or MV biogenesis in airway epithelial cells in response to mycobacterial infection in cell culture and animal models. It would be important to understand the mechanism by which *M. ab* proteins are trafficked into MVs in human or mouse bronchial epithelial cells, and determine if macrophages and bronchial epithelial cells have a distinct or similar mechanism for *M. ab* protein trafficking into MVs in the future study.

We also realized the limitation of the study. Specifically, the use of nonpolarized human bronchial epithelial cells and THP-1-derived macrophages in cell culture restricts the physiological relevance of our findings. To better reflect in vivo conditions, future studies should involve MVs isolated from polarized human bronchial epithelial cells, such as mucociliary-differentiated bronchial epithelial cell lines. These models would more accurately mimic the airway environment in animal models or humans. In particular, the ability to isolate MVs that are released either basolaterally or apically by polarized epithelial cells could provide valuable insights into the distinct roles that these two types of MVs might play in macrophage activation. Furthermore, it would be beneficial to explore the effects of MVs released from human bronchial epithelial cells on primary human macrophages, including alveolar macrophages and monocyte-derived macrophages from human peripheral blood mononuclear cells (PBMCs).

In summary, this is the first study to attempt to understand the mechanism by which human bronchial epithelial cells communicate with airway macrophages via releasing MVs in response to mycobacterial infection. It elucidates an engagement of human bronchial epithelial cell-derived MVs in the polarization of human THP-1-derived macrophages, and MV-carried ICAM-1 in regulating MV internalization by macrophages via receptor–ligand interactions in cell culture. Future studies are needed to understand the mechanism of how human bronchial epithelial cell-derived MVs regulate cellular response in macrophages. Overall, our study underscores the significance of human bronchial epithelial cell-derived MVs as key players in macrophage activation and polarization, shedding light on the development of a novel host-derived therapy for mycobacterial lung infection.

## Data Availability

The mass spectrometry proteomics data have been deposited to the ProteomeXchange Consortium via the PRIDE^18^ partner repository with the data set identifier PXD056025

## Supporting Information Available

Description for tables (PDF)
Table S1. Human proteins unique in MVs isolated from uninfected or *M. ab*-infected 16HBE14o- human bronchial epithelial cells (XLSX)
Table S2. Differentially regulated human proteins in MVs isolated from uninfected or *M. ab*-infected 16HBE14o- human bronchial epithelial cells (XLSX)
Table S3. Human proteins in top 20 upregulated pathways in MVs isolated from *M. ab*-infected 16HBE14o- human bronchial epithelial cells (XLSX)
Table S4. Human proteins in top 20 downregulated pathways in MVs isolated from *M. ab*-infected 16HBE14o- human bronchial epithelial cells (XLSX)
Table S5. M.ab proteins identified in MVs isolated from *M. ab*-infected 16HBE14o- human bronchial epithelial cells (XLSX)
Table S6. M.ab proteins in top 25 enriched pathways in MVs isolated from *M. ab*-infected 16HBE14o- human bronchial epithelial cells (XLSX)

## Notes

Statistical Analysis: Data were analyzed using PRISM GraphPad (Version 9.5.0) via one-way ANOVA, followed by Tukey’s post hoc test; *p* ≤ 0.05 was considered significant.
