# Supplementary material for: Microvesicles Derived from Human Bronchial Epithelial Cells Regulate Macrophage Activation During Mycobacterium abscessus Infection
Source: J Proteome Res. 2025 Mar 28;24(5):2291–301. doi: 10.1021/acs.jproteome.4c00827 (PMC12053935; doi:10.1021/acs.jproteome.4c00827)
Supplement: Supplementary file 1 — pr4c00827_si_001.pdf [file pr4c00827_si_001.pdf]

Supporting Information for

Microvesicles Derived from Human Bronchial Epithelial Cells Regulate Macrophage Activation During *Mycobacterium abscessus* Infection

Carlyn M. Guthrie [1,2,†], Amber C. Meeker [1,2,†], Ashton E. Self [1,2,†], Aidaly Ramos-Leyva [1,2], Olivia L. Clark [1,2], Stephen K. Kotey[1,2], Steven D. Hartson [1,4], Yurong Liang [2,3], Lin Liu [2,3], Xuejuan Tan [1,2] and Yong Cheng [1,2,*]

[1] Department of Biochemistry and Molecular Biology, Oklahoma State University, Stillwater, Oklahoma, 74078.

[2] Oklahoma Center for Respiratory and Infectious Diseases, Oklahoma State University, Stillwater, Oklahoma, 74078.

[3] Department of Physiological Sciences, Oklahoma State University, Stillwater, Oklahoma, 74078.

[4] Center for Genomics and Proteomics, Oklahoma State University, Stillwater, Oklahoma, 74078.

* Corresponding author: ycheng@okstate.edu

Supplementary Tables

1. Supplementary_Table_1.xlsx: Human proteins unique in MVs isolated from uninfected or *M.ab*-infected 16HBE14o- human bronchial epithelial cells.
2. Supplementary_Table_2.xlsx: Differentially regulated human proteins in MVs isolated from uninfected or *M.ab*-infected 16HBE14o- human bronchial epithelial cells.
3. Supplementary_Table_3.xlsx: Human proteins in top 20 upregulated pathways in MVs isolated from *M.ab*-infected 16HBE14o- human bronchial epithelial cells.
4. Supplementary_Table_4.xlsx: Human proteins in top 20 downregulated pathways in MVs isolated from *M.ab*-infected 16HBE14o- human bronchial epithelial cells.
5. Supplementary_Table_5.xlsx: *M.ab* proteins identified in MVs isolated from *M.ab*-infected 16HBE14o- human bronchial epithelial cells.
6. Supplementary_Table_6.xlsx: *M.ab* proteins in top 25 enriched pathways in MVs isolated from *M.ab*-infected 16HBE14o- human bronchial epithelial cells.
